# Supplementary material for: Adjusting agricultural emissions for trade matters for climate change mitigation
Source: Nat Commun. 2022 Jun 9;13:3024. doi: 10.1038/s41467-022-30607-x (PMC9184627; doi:10.1038/s41467-022-30607-x)
Supplement: Supplementary file 1 — Supplementary Information [file 41467_2022_30607_MOESM1_ESM.pdf]

# Supplementary Information: Adjusting agricultural emissions for trade matters for climate change mitigation

Adrian Foong [1,2,3], Prajal Pradhan [1,*], Oliver Frör [2], Jürgen P. Kropp [1,4]

[1] Potsdam Institute for Climate Impact Research (PIK), Member of the Leibniz Association, P.O. Box 60 12 03, D-14412 Potsdam, Germany

[2] University of Koblenz-Landau, Institute for Environmental Sciences, Landau, Germany

[3] adelphi research gemeinnützige GmbH, Berlin, Germany[4] University of Potsdam, Institute for Environmental Science and Geography, Potsdam, Germany

*pradhan@pik-potsdam.de

## Supplementary Figures

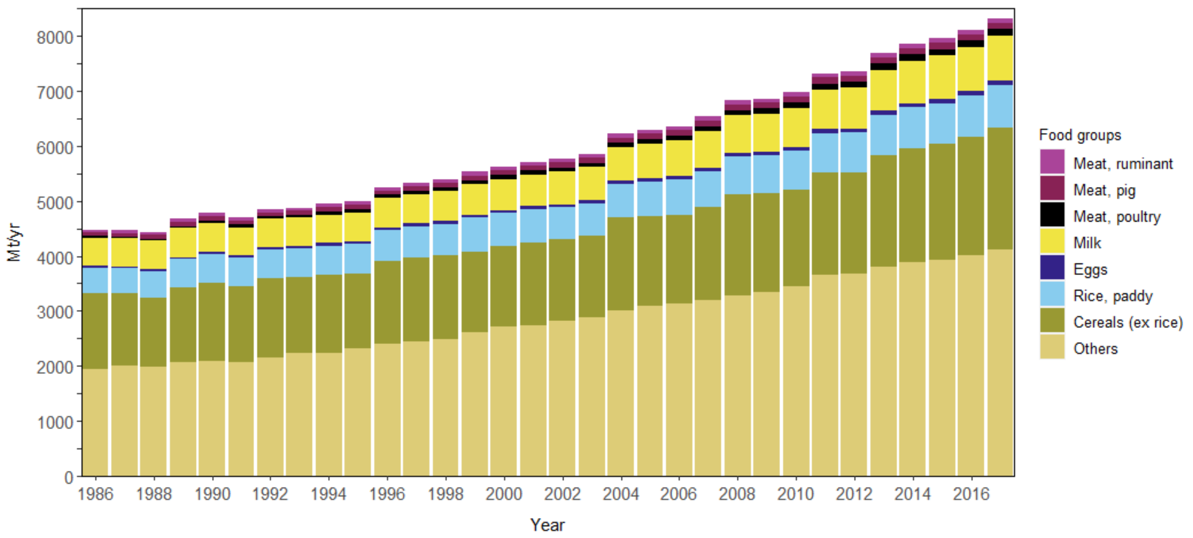


***Supplementary Figure 1: Global change in agricultural production volumes from 1986 to 2017.*** *Production volumes are broken down according to food groups. Values are shown for individual years, i.e., without averaging values for three years.*

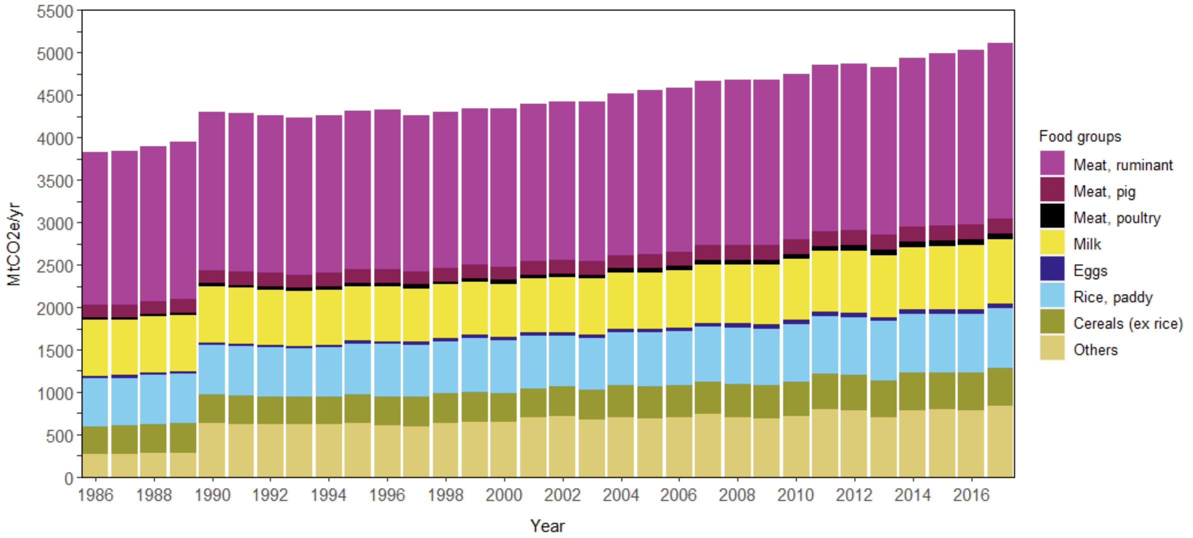


***Supplementary Figure 2: Global change in production-based emissions (PBEs) from 1986 to 2017.*** *PBEs are broken down according to food groups. Values are shown for individual years, i.e., without averaging values for three years.*

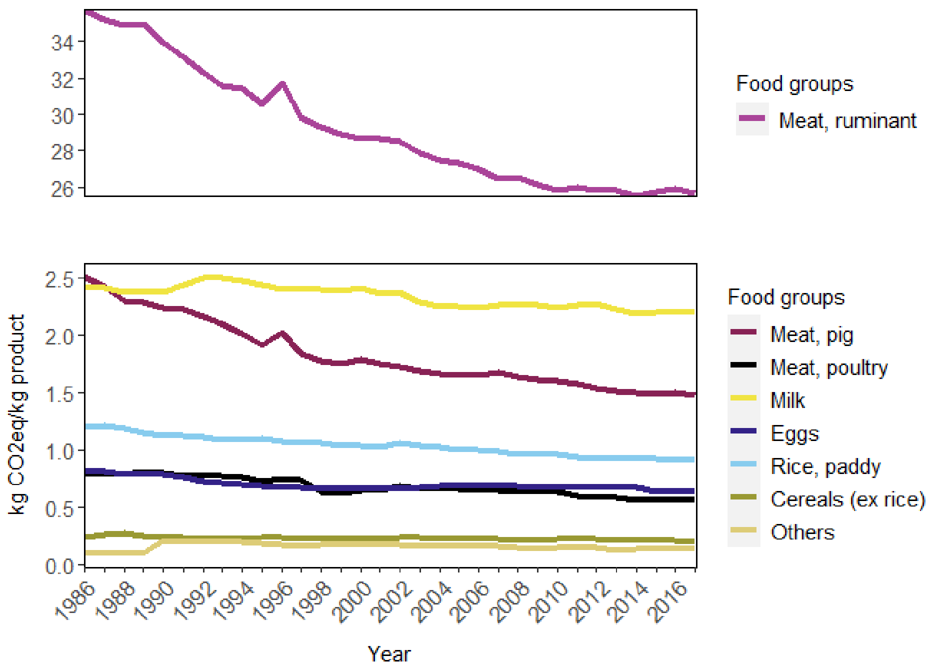


***Supplementary Figure 3: Change in global-averaged emission intensities of eight food groups from 1986 to 2017.*** *Note the different y-axis scale used for the food group 'Meat, ruminant', the emission intensity of which is more than ten times larger than those of all other food groups in all years. The jump in emission intensity of 'others' between 1989 and 1990 is due to the spike in total agricultural emissions for most countries in the FAOSTAT dataset 'Agriculture Total' during the same period. Values are shown for individual years, i.e., without averaging values for three years.*

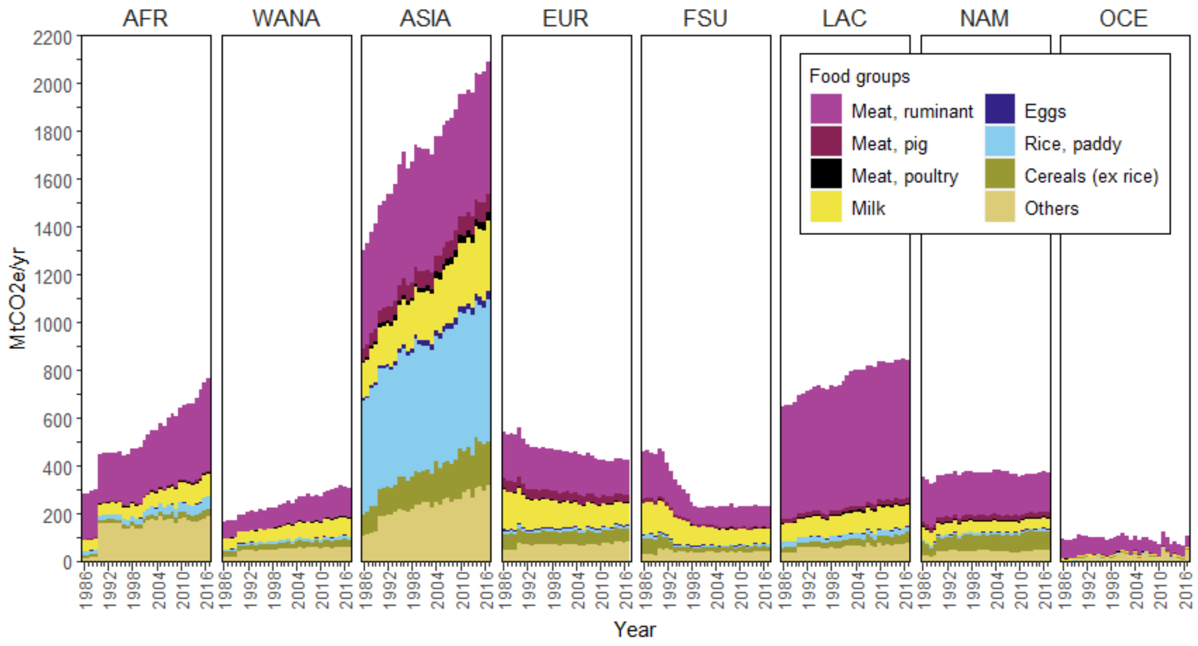


***Supplementary Figure 4: Regional change in trade-adjusted agricultural emissions (TAEs) from 1986 to 2017.*** *The regions analysed in this study are Africa (AFR), Western Asia and Northern Africa (WANA), Asia (ASIA), Europe (EUR), Former Soviet Union (FSU), Latin America and the Caribbean (LAC), North America (NAM), and Oceania (OCE). Additionally, the figure also provides a breakdown of the share of each food group. Values are shown for individual years, i.e., without averaging values for three years.*

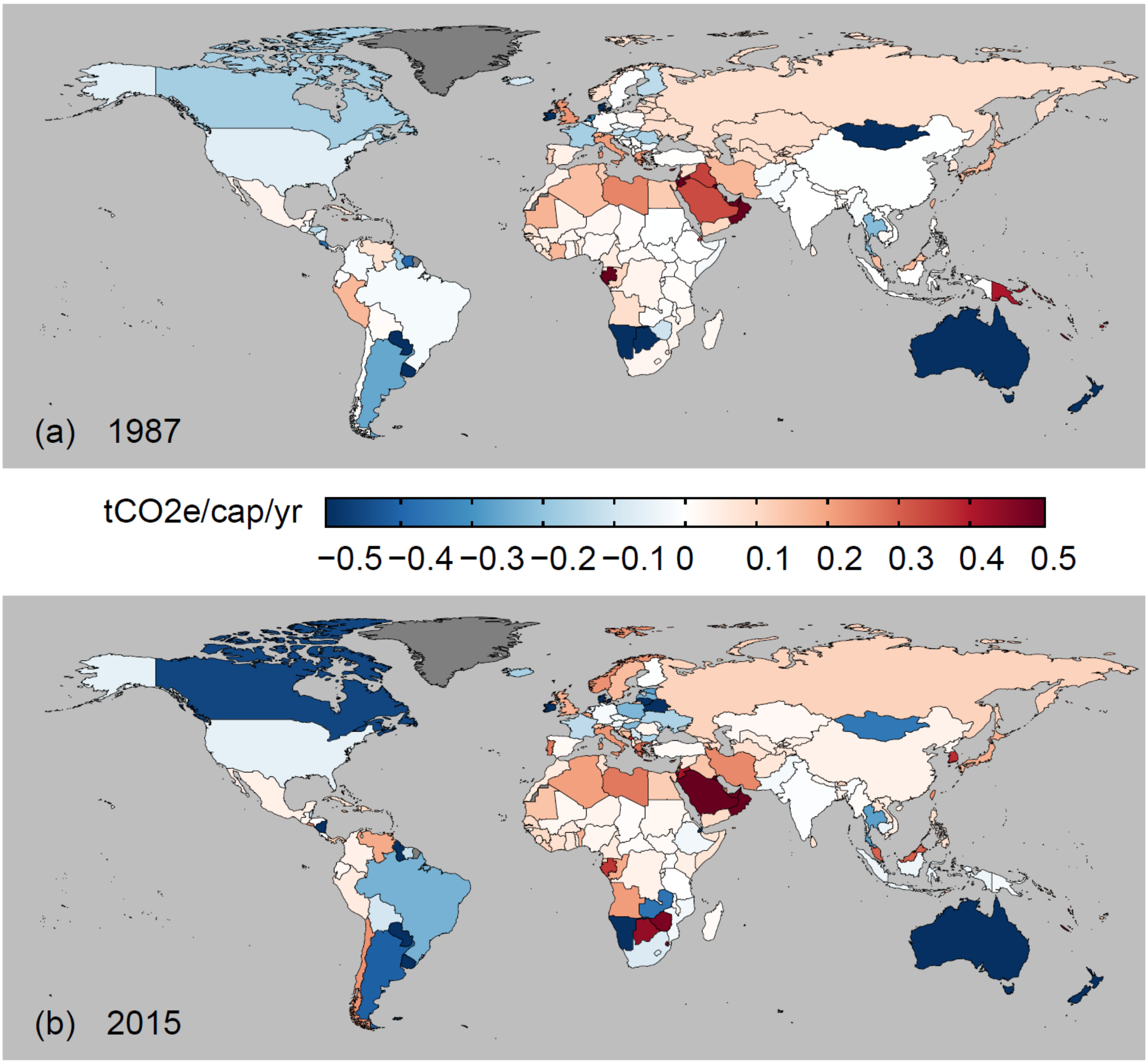


***Supplementary Figure 5: Per capita differences between trade-adjusted agricultural emissions (TAEs) and production-based emissions (PBEs) in 1987 (a) and 2015 (b).*** *Differences are calculated by subtracting PBE per capita from TAE per capita, such that the larger the positive difference (darker red), the higher is a country's per capita TAE compared to its per capita PBE (i.e., net importers of agricultural emissions). Likewise, the larger the negative difference (darker blue), the lower is a country's per capita TAE than its per capita PBE (i.e., net exporters of agricultural emissions). Dark grey colours indicate countries with no available data (e.g., Greenland).*

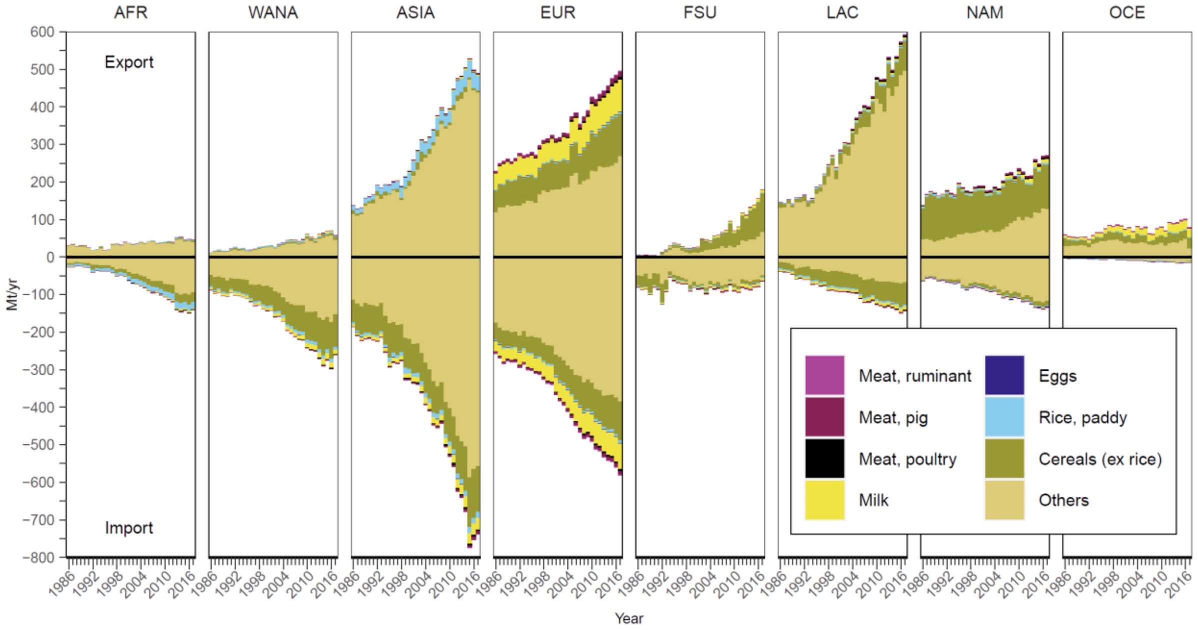


***Supplementary Figure 6: Regional change in export and import volumes from 1986 to 2017**. Exports are shown at the top half of the figure, while imports are shown at the bottom half. The regions analysed in this study are Africa (AFR), Western Asia and Northern Africa (WANA), Asia (ASIA), Europe (EUR), Former Soviet Union (FSU), Latin America and the Caribbean (LAC), North America (NAM), and Oceania (OCE). Additionally, the figure also provides a breakdown of the share of each food group. Values are shown for individual years, i.e., without averaging values for three years.*

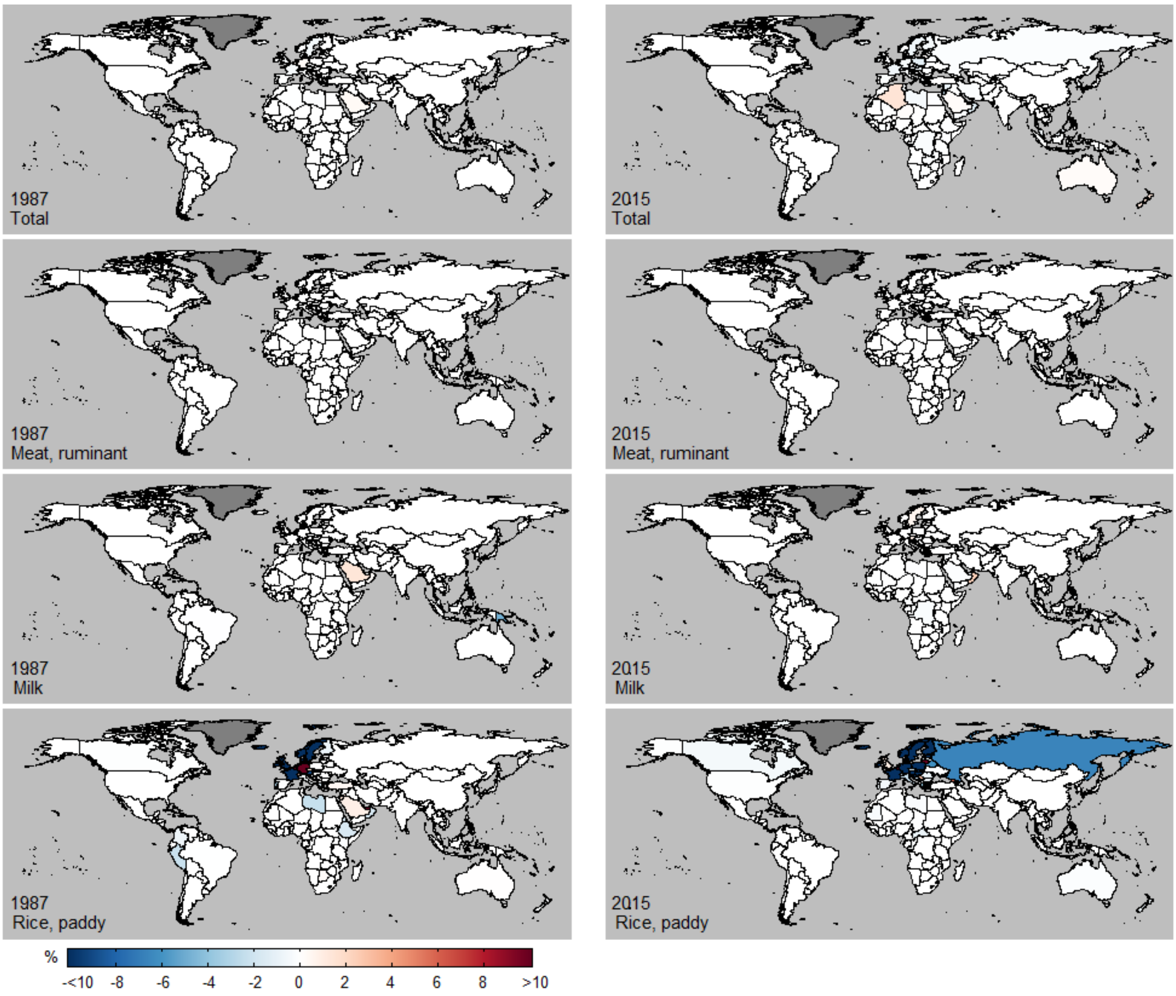


***Supplementary Figure 7: Comparison of trade-adjusted agricultural emissions (TAEs) between original method and sensitivity analysis in which regional emission intensities were replaced by global emission intensities for non-producer countries, in 1987 and 2015***. *Comparison is made by calculating the percentage weighted differences between the TAEs of both approaches. Positive (darker red) values indicate lower TAEs using original method, whereas negative (darker blue) values indicate higher TAEs using original method. For example, the dark blue colours of Europe and Russia for paddy rice (bottom right panel) show that TAEs for paddy rice are higher using the original method, thus indicating the higher emission intensities in each respective region compared to the global average emission intensity for paddy rice. Dark grey colours indicate countries with no available data (e.g., Greenland).*

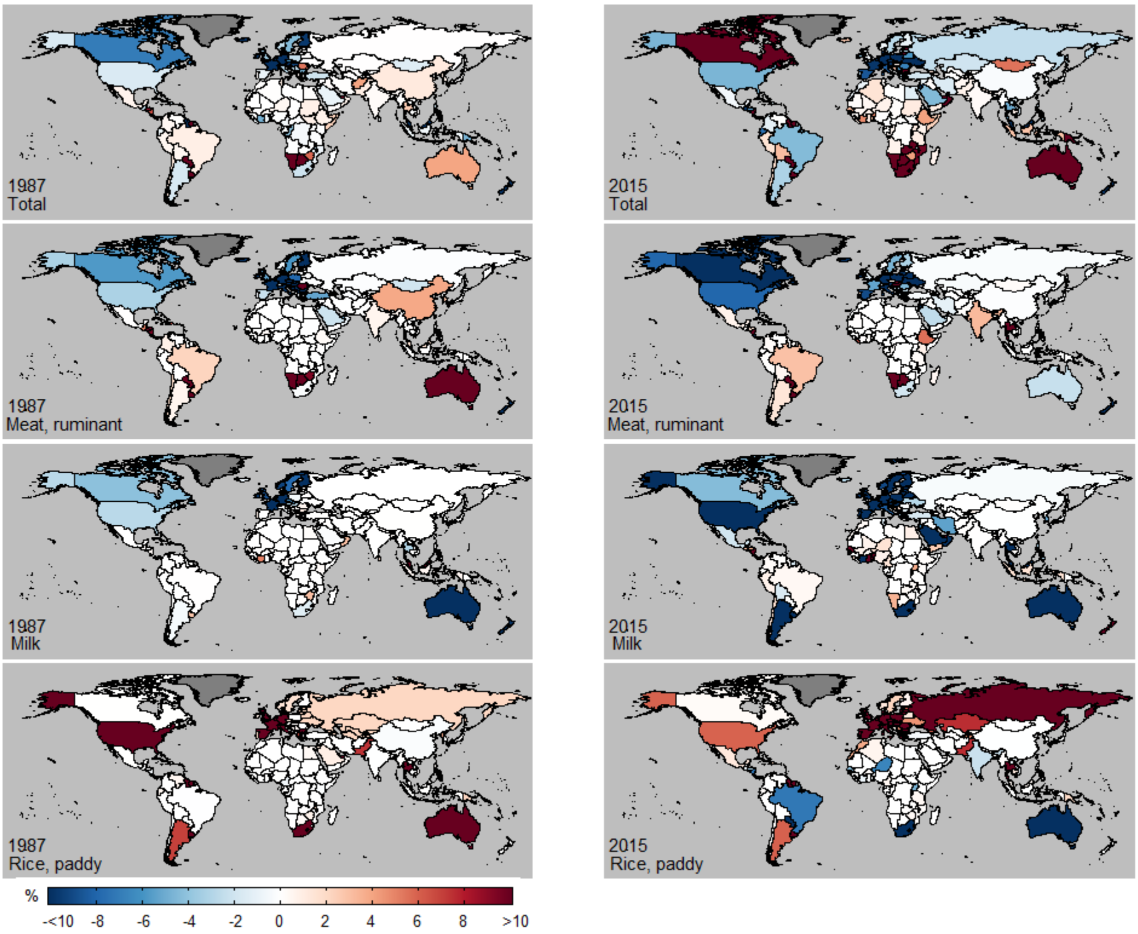


***Supplementary Figure 8: Comparison of trade-adjusted agricultural emissions (TAEs) between original method and sensitivity analysis in which a 'technology-adjusted' approach based on Kander et al. (2015) was adopted, in 1987 and 2015**. Comparison is made by calculating the percentage weighted differences between the TAEs of both approaches. Positive (darker red) values indicate lower TAEs using original method, whereas negative (darker blue) values indicate higher TAEs using original method. Dark grey colours indicate countries with no available data (e.g., Greenland).*

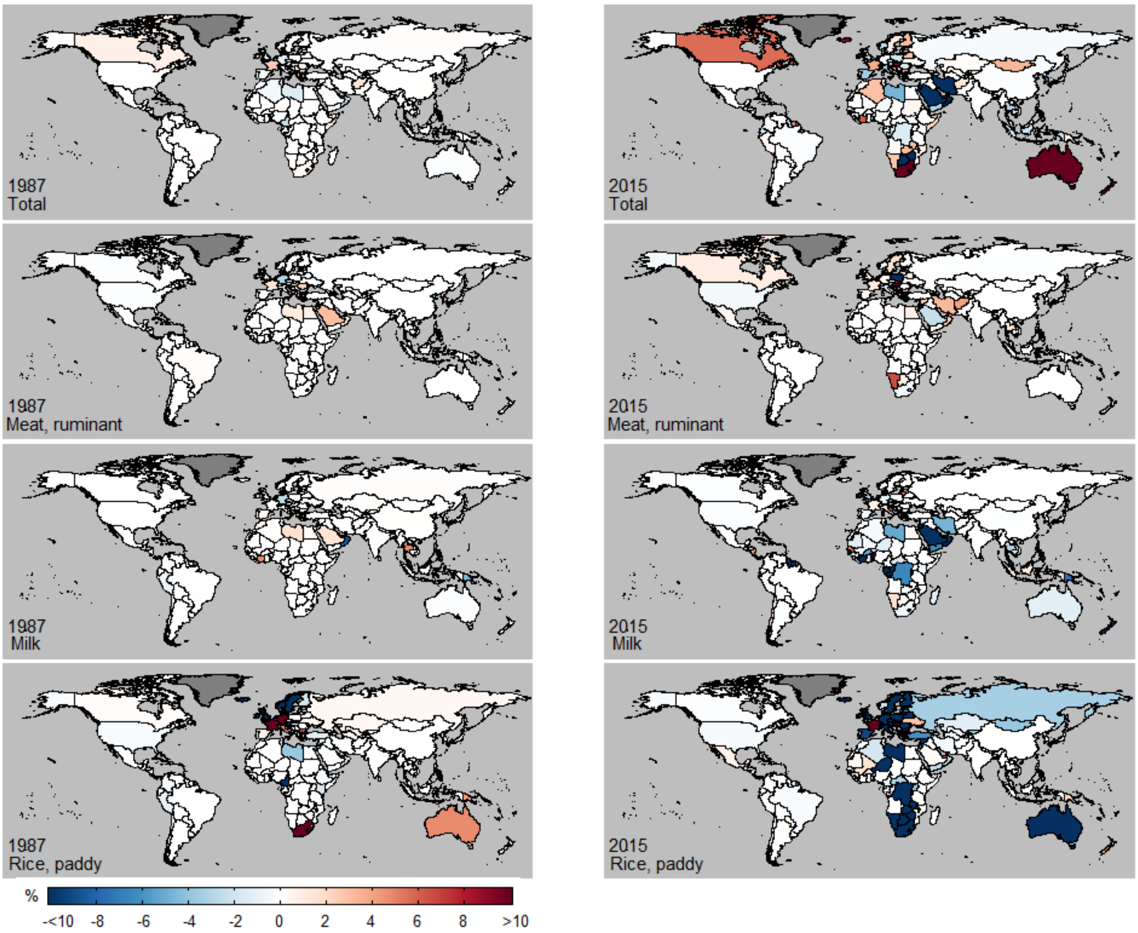


***Supplementary Figure 9: Comparison of trade-adjusted agricultural emissions (TAEs) between original method and sensitivity analysis in which emission intensities for exports are adjusted to account for both production and imports, in 1987 and 2015****. Comparison is made by calculating the percentage weighted differences between the TAEs of both approaches. Positive (darker red) values indicate lower TAEs using original method, whereas negative (darker blue) values indicate higher TAEs using original method. Dark grey colours indicate countries with no available data (e.g., Greenland).*

.

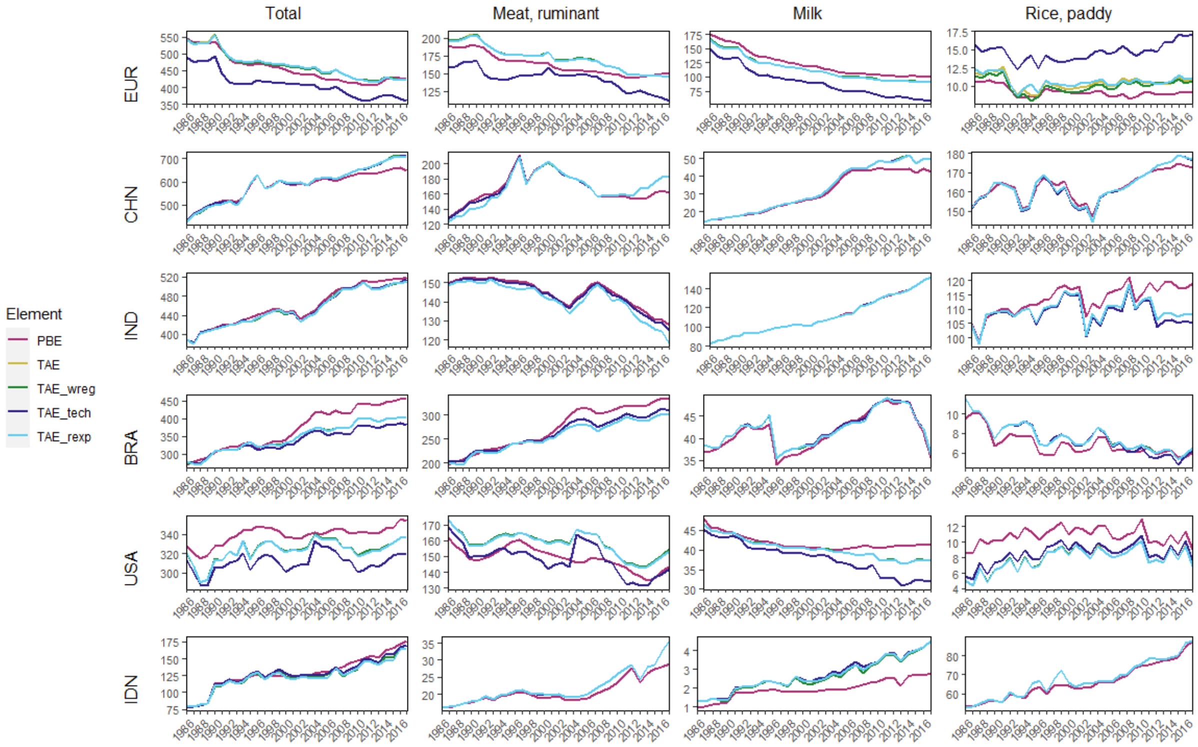


***Supplementary Figure 10: Changes in production-based emissions (PBEs) and trade-adjusted agricultural emissions (TAEs) from 1986 to 2017.*** *TAEs are shown for (1) the original approach, (2) the sensitivity analysis in which global emission intensities were used (TAE_wreg), (3) the sensitivity analysis which adopted a 'technology-adjusted' approach (TAE_tech), and (4) the sensitivity analysis which used emission intensities that account for both production and imports (TAE_rexp). The region/countries shown are Europe (EUR), China, mainland (CHN), India (IND), Brazil (BRA). United States of America (USA), and Indonesia (IDN). These are the five countries with the largest total absolute TAEs in 2015, while EUR is shown to provide a regional perspective. PBEs and TAEs are shown for all food groups collectively (Total), as well as for three food groups, i.e., (1) meat, ruminant, (2) milk, and (3) rice, paddy, which represent the food groups with the largest emission intensities. Values are shown for individual years, i.e., without averaging values for three years.*

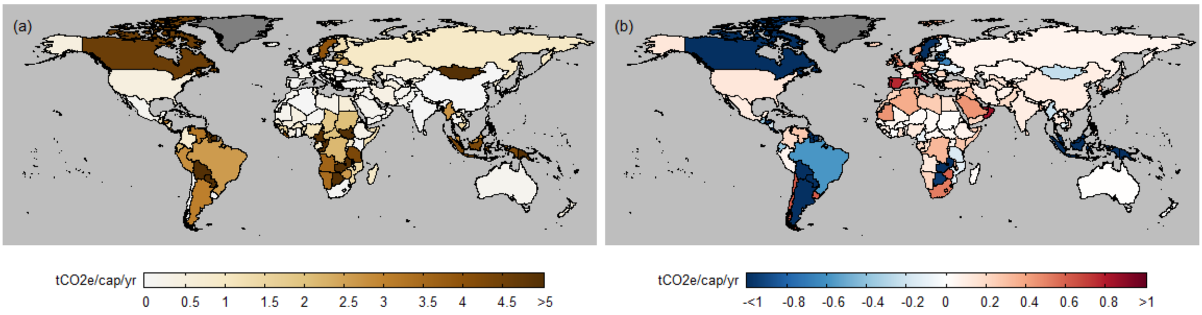


***Supplementary Figure 11: Agricultural land use emissions per capita for 2015****. Left panel (a) shows agricultural land use emissions per capita without trade adjustments. Right panel (b) shows the per capita differences between trade-adjusted agricultural land use emissions and agricultural land use emissions. Differences are calculated such that the larger the positive difference (darker red), the higher is a country's trade-adjusted agricultural land use emissions per capita (i.e., net importers of agricultural land use emissions). Likewise, the larger the negative difference (darker blue), the lower is a country's trade-adjusted agricultural land use emissions per capita (i.e., net exporters of agricultural land use emissions). Dark grey colours indicate countries with no available data (e.g., Greenland).*

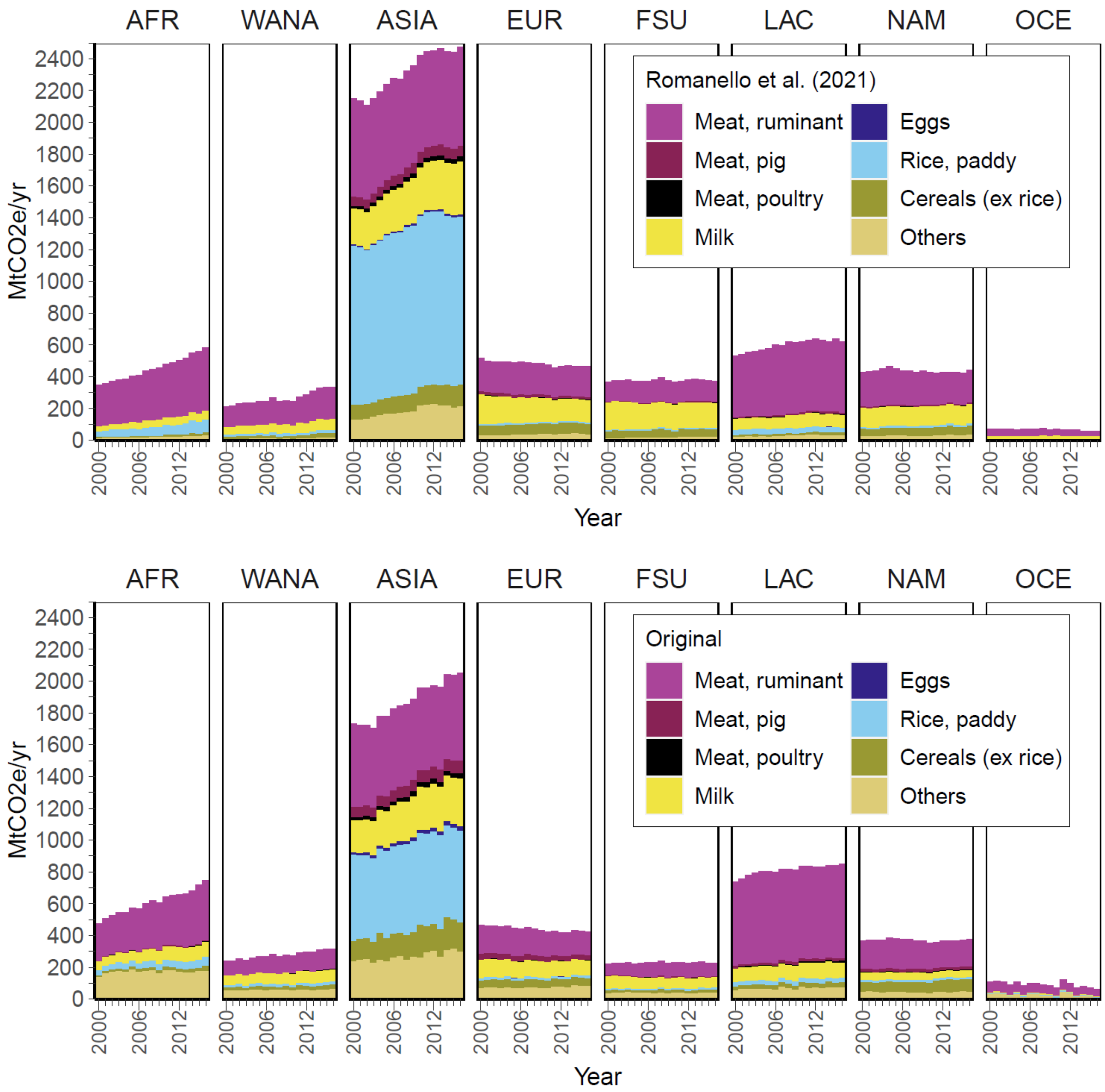


***Supplementary Figure 12: Regional change in trade-adjusted agricultural emissions (TAEs) from 2000 to 2016, based on the approach used by Romanello et al. (2021) (above) and our approach (below).*** *The regions analysed are Africa (AFR), Western Asia and Northern Africa (WANA), Asia (ASIA), Europe (EUR), Former Soviet Union (FSU), Latin America and the Caribbean (LAC), North America (NAM), and Oceania (OCE). Additionally, the figure also provides a breakdown of the share of each food group. Values are shown for individual years, i.e., without averaging values for three years.*

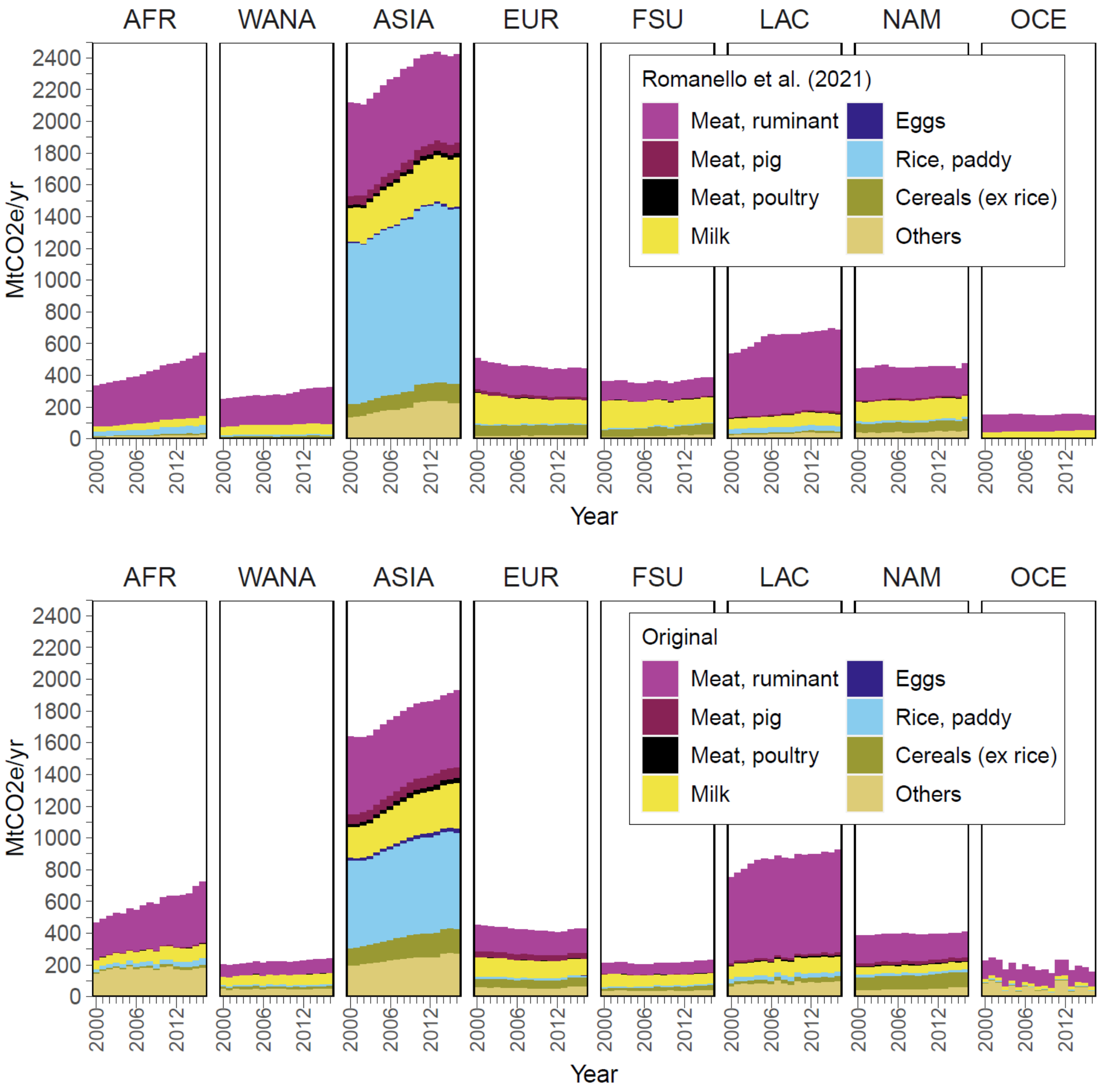


***Supplementary Figure 13: Regional change in production-based emissions (PBEs) from 2000 to 2016, based on the approach used by Romanello et al. (2021) (above) and our approach (below).*** *The regions analysed are Africa (AFR), Western Asia and Northern Africa (WANA), Asia (ASIA), Europe (EUR), Former Soviet Union (FSU), Latin America and the Caribbean (LAC), North America (NAM), and Oceania (OCE). Additionally, the figure also provides a breakdown of the share of each food group. Values are shown for individual years, i.e., without averaging values for three years.*

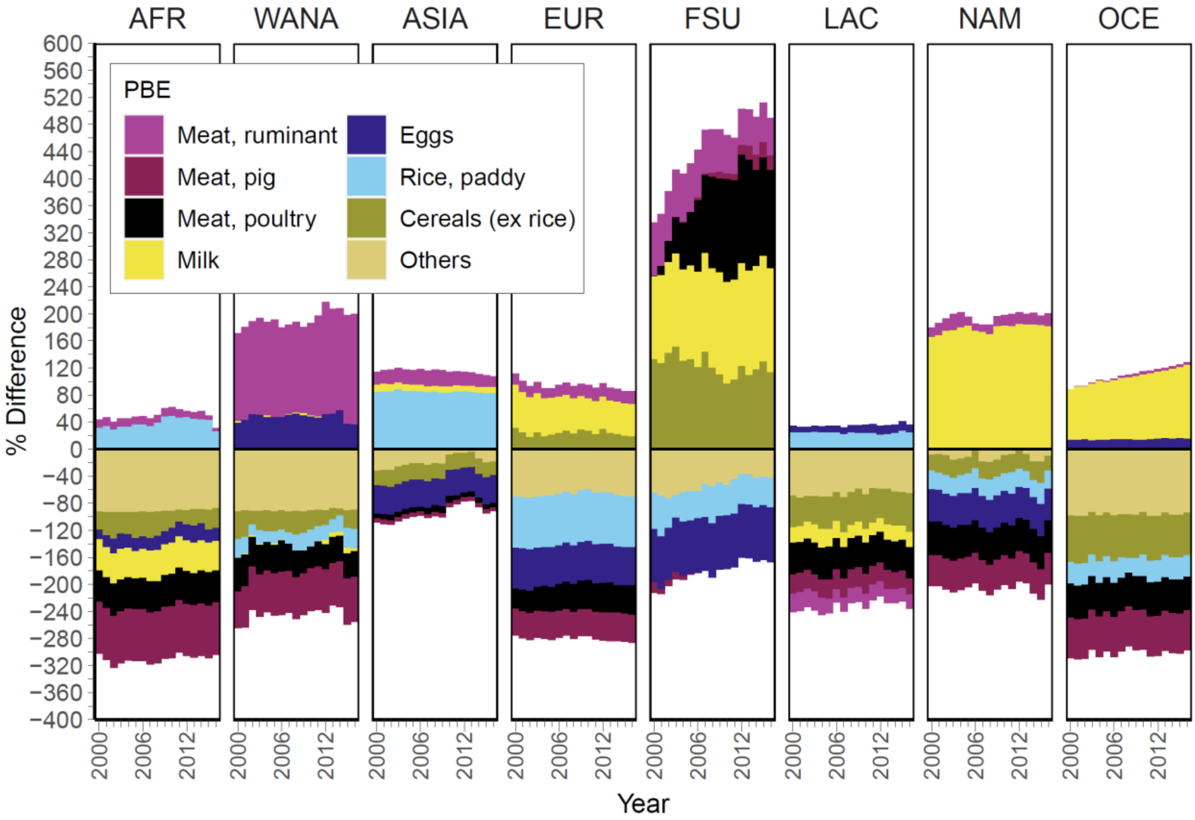


***Supplementary Figure 14: Percentage relative differences between production-based emissions (PBEs) from the approach used by Romanello et al. (2021) and our approach, from 2000 to 2016.*** *Positive values indicate larger PBEs in Romanello et al. (2021) compared to our approach, whereas negative values indicate otherwise. The regions analysed are Africa (AFR), Western Asia and Northern Africa (WANA), Asia (ASIA), Europe (EUR), Former Soviet Union (FSU), Latin America and the Caribbean (LAC), North America (NAM), and Oceania (OCE). Additionally, the figure also provides a breakdown of the share of each food group. Values are shown for individual years, i.e., without averaging values for three years.*

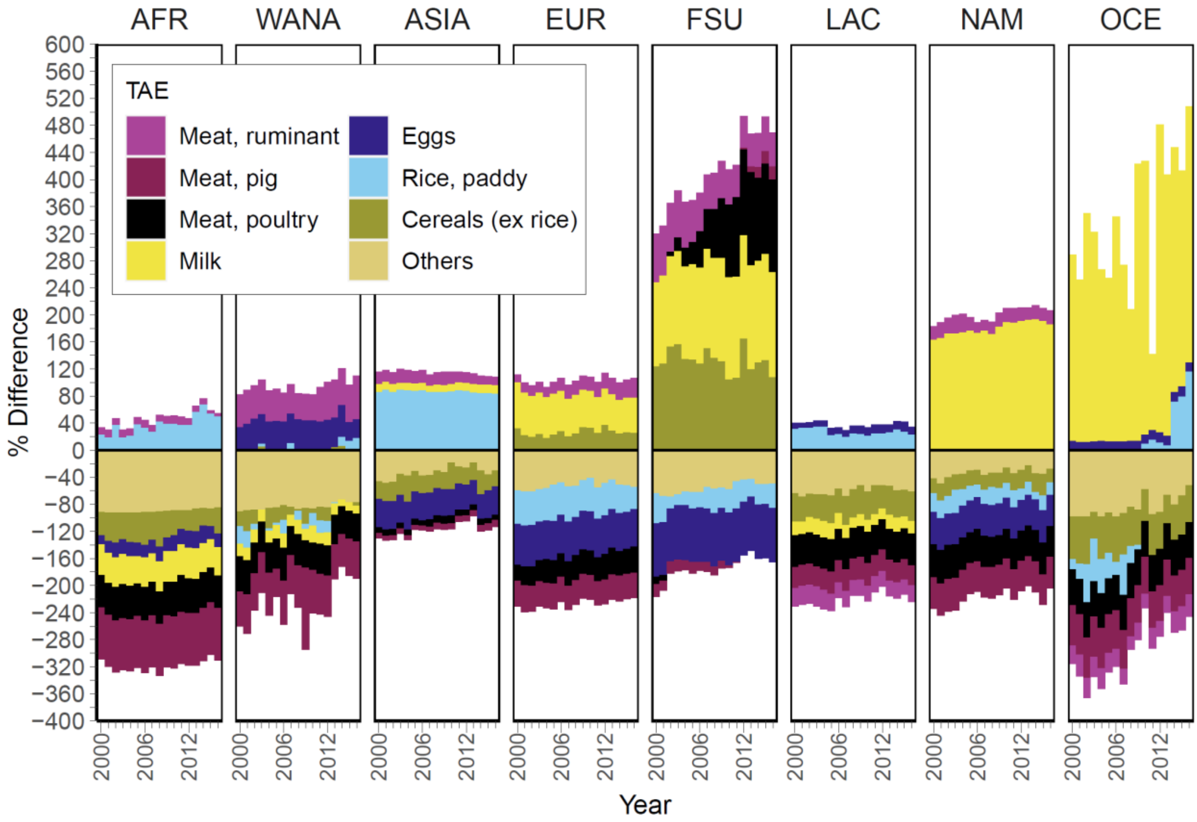


***Supplementary Figure 15: Percentage relative differences between trade-adjusted agricultural emissions (TAEs) from the approach used by Romanello et al. (2021) and our approach, from 2000 to 2016.*** *Positive values indicate larger TAEs in Romanello et al. (2021) compared to our approach, whereas negative values indicate otherwise. The regions analysed are Africa (AFR), Western Asia and Northern Africa (WANA), Asia (ASIA), Europe (EUR), Former Soviet Union (FSU), Latin America and the Caribbean (LAC), North America (NAM), and Oceania (OCE). Additionally, the figure also provides a breakdown of the share of each food group. Values are shown for individual years, i.e., without averaging values for three years.*

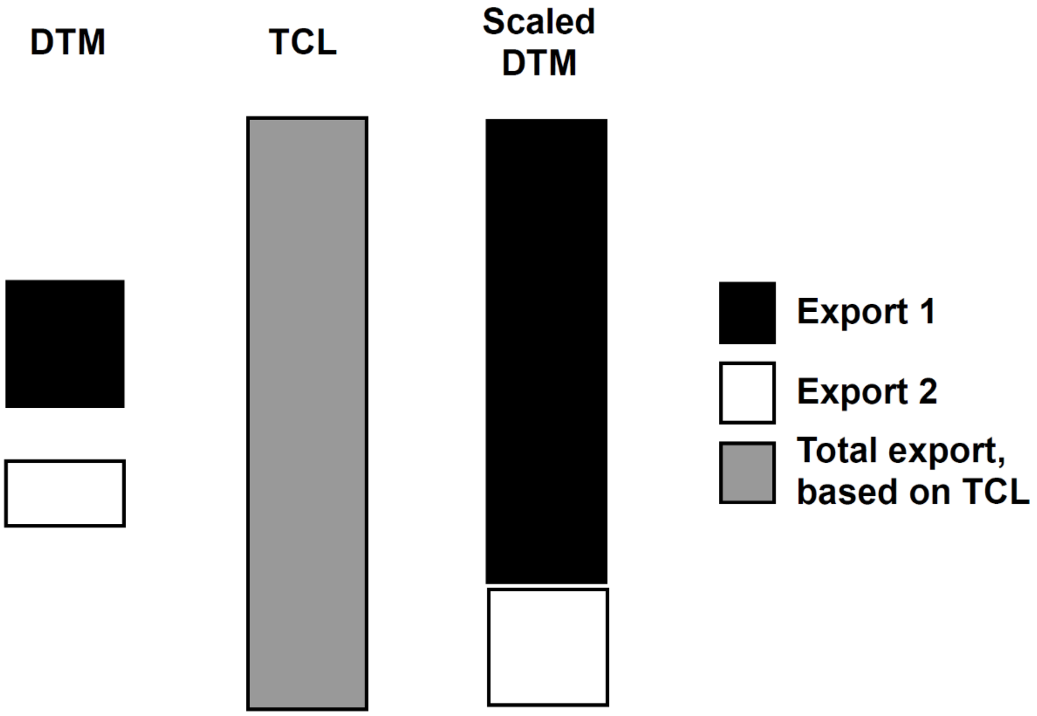


***Supplementary Figure 16: Illustration of the trade data scaling procedure used in this study.*** *This procedure harmonises export and import figures from FAOSTAT's 'Detailed Trade Matrix' (DTM) and 'Trade – Crops and Livestock' (TCL) datasets, such that the sum of exports in the DTM matches those recorded in the TCL.*

## Supplementary Tables

***Supplementary Table 1: List of countries and their respective UN ISO 3166-1 alpha-3 code and regional group.*** *Groups are loosely based on the UN M49 Standard Geographical Classification[1]. These regions are Africa (AFR), Asia (ASIA), Former Soviet Union (FSU), Europe (EUR), Latin America and the Caribbean (LAC), Western Asia and Northern Africa (WANA), North America (NAM), and Oceania (OCE).*

| Country | ISO 3166-1 alpha-3 code | Region |
|---|---|---|
| Afghanistan | AFG | ASIA |
| Albania | ALB | EUR |
| Algeria | DZA | WANA |
| American Samoa | ASM | OCE |
| Angola | AGO | AFR |
| Antigua and Barbuda | ATG | LAC |
| Argentina | ARG | LAC |
| Armenia | ARM | FSU |
| Australia | AUS | OCE |
| Austria | AUT | EUR |
| Azerbaijan | AZE | FSU |
| Bahamas | BHS | LAC |
| Bahrain | BHR | WANA |
| Bangladesh | BGD | ASIA |
| Barbados | BRB | LAC |
| Belarus | BLR | FSU |
| Belgium | BEL | EUR |
| Belgium-Luxembourg[2] | - | EUR |
| Belize | BLZ | LAC |
| Benin | BEN | AFR |
| Bermuda | BMU | NAM |
| Bhutan | BTN | ASIA |
| Bolivia (Plurinational State of) | BOL | LAC |
| Bosnia and Herzegovina | BIH | EUR |
| Botswana | BWA | AFR |
| Brazil | BRA | LAC |
| British Virgin Islands | VGB | LAC |
| Brunei Darussalam | BRN | ASIA |
| Bulgaria | BGR | EUR |
| Burkina Faso | BFA | AFR |
| Burundi | BDI | AFR |
| Cabo Verde | CPV | AFR |
| Cambodia | KHM | ASIA |
| Cameroon | CMR | AFR |
| Canada | CAN | NAM |
| Cayman Islands | CYM | LAC |
| Central African Republic | CAF | AFR |

[1] UN Statistics 2020: Methodology: Standard country or area codes for statistical use (M49), URL: https://unstats.un.org/unsd/methodology/m49/, accessed on 16.03.2020.

[2] Not a country *per se*, but rather an economic union between Belgium and Luxembourg, the name of which was used to represent both countries collectively for international trade statistics prior to 1999.

| Country | ISO 3166-1 alpha-3 code | Region |
|---|---|---|
| Chad | TCD | AFR |
| Chile | CHL | LAC |
| China, Hong Kong SAR | HKG | ASIA |
| China, Macao SAR | MAC | ASIA |
| China, mainland | CHN | ASIA |
| China, Taiwan Province of | TWN | ASIA |
| Colombia | COL | LAC |
| Comoros | COM | AFR |
| Congo | COG | AFR |
| Cook Islands | COK | OCE |
| Costa Rica | CRI | LAC |
| Côte d'Ivoire | CIV | AFR |
| Croatia | HRV | EUR |
| Cuba | CUB | LAC |
| Cyprus | CYP | WANA |
| Czechia | CZE | EUR |
| Czechoslovakia | CSK | EUR |
| Democratic People's Republic of Korea | PRK | ASIA |
| Democratic Republic of the Congo | COD | AFR |
| Denmark | DNK | EUR |
| Djibouti | DJI | AFR |
| Dominica | DMA | LAC |
| Dominican Republic | DOM | LAC |
| Ecuador | ECU | LAC |
| Egypt | EGY | WANA |
| El Salvador | SLV | LAC |
| Equatorial Guinea | GNQ | AFR |
| Eritrea | ERI | AFR |
| Estonia | EST | FSU |
| Eswatini | SWZ | AFR |
| Ethiopia | ETH | AFR |
| Ethiopia PDR | ETH | AFR |
| Falkland Islands (Malvinas) | FLK | LAC |
| Faroe Islands | FRO | EUR |
| Fiji | FJI | OCE |
| Finland | FIN | EUR |
| France | FRA | EUR |
| French Polynesia | PYF | OCE |
| Gabon | GAB | AFR |
| Gambia | GMB | AFR |
| Georgia | GEO | FSU |
| Germany | DEU | EUR |
| Ghana | GHA | AFR |
| Greece | GRC | EUR |
| Grenada | GRD | LAC |
| Guam | GUM | OCE |
| Guatemala | GTM | LAC |

| Country | ISO 3166-1 alpha-3 code | Region |
|---|---|---|
| Guinea | GIN | AFR |
| Guinea-Bissau | GNB | AFR |
| Guyana | GUY | LAC |
| Haiti | HTI | LAC |
| Honduras | HND | LAC |
| Hungary | HUN | EUR |
| Iceland | ISL | EUR |
| India | IND | ASIA |
| Indonesia | IDN | ASIA |
| Iran (Islamic Republic of) | IRN | ASIA |
| Iraq | IRQ | WANA |
| Ireland | IRL | EUR |
| Israel | ISR | WANA |
| Italy | ITA | EUR |
| Jamaica | JAM | LAC |
| Japan | JPN | ASIA |
| Jordan | JOR | WANA |
| Kazakhstan | KAZ | FSU |
| Kenya | KEN | AFR |
| Kiribati | KIR | OCE |
| Kuwait | KWT | WANA |
| Kyrgyzstan | KGZ | FSU |
| Lao People's Democratic Republic | LAO | ASIA |
| Latvia | LVA | FSU |
| Lebanon | LBN | WANA |
| Lesotho | LSO | AFR |
| Liberia | LBR | AFR |
| Libya | LBY | WANA |
| Lithuania | LTU | FSU |
| Luxembourg | LUX | EUR |
| Madagascar | MDG | AFR |
| Malawi | MWI | AFR |
| Malaysia | MYS | ASIA |
| Maldives | MDV | ASIA |
| Mali | MLI | AFR |
| Malta | MLT | EUR |
| Mauritania | MRT | AFR |
| Mauritius | MUS | AFR |
| Mexico | MEX | LAC |
| Mongolia | MNG | ASIA |
| Montenegro | MNE | EUR |
| Morocco | MAR | WANA |
| Mozambique | MOZ | AFR |
| Myanmar | MMR | ASIA |
| Namibia | NAM | AFR |
| Nauru | NRU | OCE |
| Nepal | NPL | ASIA |

| Country | ISO 3166-1 alpha-3 code | Region |
|---|---|---|
| Netherlands | NLD | EUR |
| Netherlands Antilles (former) | ANT | LAC |
| New Caledonia | NCL | OCE |
| New Zealand | NZL | OCE |
| Nicaragua | NIC | LAC |
| Niger | NER | AFR |
| Nigeria | NGA | AFR |
| Niue | NIU | OCE |
| North Macedonia | MKD | EUR |
| Norway | NOR | EUR |
| Palestine | PSE | WANA |
| Oman | OMN | WANA |
| Pacific Islands Trust Territory | PCI | OCE |
| Pakistan | PAK | ASIA |
| Panama | PAN | LAC |
| Papua New Guinea | PNG | OCE |
| Paraguay | PRY | LAC |
| Peru | PER | LAC |
| Philippines | PHL | ASIA |
| Poland | POL | EUR |
| Portugal | PRT | EUR |
| Qatar | QAT | WANA |
| Republic of Korea | KOR | ASIA |
| Republic of Moldova | MDA | FSU |
| Romania | ROU | EUR |
| Russian Federation | RUS | FSU |
| Rwanda | RWA | AFR |
| Saint Kitts and Nevis | KNA | LAC |
| Saint Lucia | LCA | LAC |
| Saint Pierre and Miquelon | SPM | NAM |
| Saint Vincent and the Grenadines | VCT | LAC |
| Samoa | WSM | OCE |
| Sao Tome and Principe | STP | AFR |
| Saudi Arabia | SAU | WANA |
| Senegal | SEN | AFR |
| Serbia | SRB | EUR |
| Serbia and Montenegro | SCG | EUR |
| Seychelles | SYC | AFR |
| Sierra Leone | SLE | AFR |
| Singapore | SGP | ASIA |
| Slovakia | SVK | EUR |
| Slovenia | SVN | EUR |
| Solomon Islands | SLB | OCE |
| Somalia | SOM | AFR |
| South Africa | ZAF | AFR |
| South Sudan | SSD | WANA |
| Spain | ESP | EUR |

| Country | ISO 3166-1 alpha-3 code | Region |
|---|---|---|
| Sri Lanka | LKA | ASIA |
| Sudan | SDN | WANA |
| Sudan (former) | SDN | WANA |
| Suriname | SUR | LAC |
| Sweden | SWE | EUR |
| Switzerland | CHE | EUR |
| Syrian Arab Republic | SYR | WANA |
| Tajikistan | TJK | FSU |
| Thailand | THA | ASIA |
| Timor-Leste | TLS | ASIA |
| Togo | TGO | AFR |
| Tonga | TON | OCE |
| Trinidad and Tobago | TTO | LAC |
| Tunisia | TUN | WANA |
| Turkey | TUR | WANA |
| Turkmenistan | TKM | FSU |
| Tuvalu | TUV | OCE |
| Uganda | UGA | AFR |
| Ukraine | UKR | FSU |
| United Arab Emirates | ARE | WANA |
| United Kingdom | GBR | EUR |
| United Republic of Tanzania | TZA | AFR |
| United States of America | USA | NAM |
| Uruguay | URY | LAC |
| USSR | SUN | FSU |
| Uzbekistan | UZB | FSU |
| Vanuatu | VUT | OCE |
| Venezuela (Bolivarian Republic of) | VEN | LAC |
| Viet Nam | VNM | ASIA |
| Yemen | YEM | WANA |
| Yugoslav SFR | YUG | EUR |
| Zambia | ZMB | AFR |
| Zimbabwe | ZWE | AFR |

***Supplementary Table 2: List of food-related agricultural items.*** *Names are shown as recorded in FAOSTAT, and the list includes each item's respective group, FAOSTAT emission intensity (EI) allocation, primary item, calorie values in kcal/100g, and source for calorie values. Sources are either from the FAO's nutritive factors list[3], or from the United States Department of Agriculture (USDA)[4]. (*) indicates items that were not analysed in the study as they do not appear in the trade datasets, but whose calorie values were needed for conversion to primary item equivalents.*

| Commodity | Group | EI | Primary item | Calories (kcal/100g) | Source |
|---|---|---|---|---|---|
| Almonds shelled | Others | Others | Almonds, with shell | 589 | FAO |
| Almonds, with shell | Others | Others | Almonds, with shell | 236 | FAO |
| Anise, badian, fennel, coriander | Others | Others | Anise, badian, fennel, coriander | 345 | FAO |
| Apples | Others | Others | Apples | 48 | FAO |
| Apricots | Others | Others | Apricots | 45 | FAO |
| Apricots, dry | Others | Others | Apricots | 238 | FAO |
| Artichokes | Others | Others | Artichokes | 20 | FAO |
| Asparagus | Others | Others | Asparagus | 12 | FAO |
| Avocados | Others | Others | Avocados | 119 | FAO |
| Bacon and ham | Meat, pig | Meat, pig | Meat, pig | 362 | FAO |
| Bambara beans | Others | Others | Bambara beans | 365 | FAO |
| Bananas | Others | Others | Bananas | 60 | FAO |
| Barley | Cereals (ex rice) | Cereals excluding rice | Barley | 332 | FAO |
| Barley, pearled | Cereals (ex rice) | Cereals excluding rice | Barley | 346 | FAO |
| Beans, dry | Others | Others | Beans, dry | 341 | FAO |
| Beans, green | Others | Others | Beans, green | 50 | FAO |
| Beer of barley | Cereals (ex rice) | Cereals excluding rice | Barley | 49 | FAO |
| Beer of sorghum | Cereals (ex rice) | Cereals excluding rice | Sorghum | 40 | FAO |
| Beverages, fermented rice | Rice, paddy | Rice, paddy | Rice, paddy | 133 | FAO |
| Blueberries | Others | Others | Blueberries | 120 | FAO |
| Bran, maize | Cereals (ex rice) | Cereals excluding rice | Maize | 224 | USDA[5] |
| Bran, wheat | Cereals (ex rice) | Cereals excluding rice | Wheat | 213 | FAO |
| Brazil nuts, shelled | Others | Others | Brazil nuts, with shell | 656 | FAO |

[3] FAO 2019: Nutritive Factors, URL: http://www.fao.org/economic/the-statistics-division-ess/publications-studies/publications/nutritive-factors/en/, accessed on 05.06.2019.
[4] USDA 2018: USDA Food Composition Databases, URL: https://ndb.nal.usda.gov/ndb/search/list?home=true, accessed on 05.06.2019.
[5] USDA name: Corn bran, crude; USDA Data Type: SR Legacy.

| Commodity | Group | EI | Primary item | Calories (kcal/100g) | Source |
|---|---|---|---|---|---|
| Brazil nuts, with shell * | Others | Others | Brazil nuts, with shell | 315 | FAO |
| Bread | Cereals (ex rice) | Cereals excluding rice | Wheat | 249 | FAO |
| Broad beans, horse beans, dry | Others | Others | Broad beans, horse beans, dry | 341 | USDA[6] |
| Buckwheat | Cereals (ex rice) | Others | Buckwheat | 330 | FAO |
| Bulgur | Cereals (ex rice) | Cereals excluding rice | Wheat | 345 | FAO |
| Butter of karite nuts | Others | Others | Karite nuts (sheanuts) | 711 | FAO |
| Butter, cow milk | Milk | Milk, whole fresh cow | Milk, whole fresh cow | 717 | FAO |
| Buttermilk, curdled, acidified milk | Milk | Milk, whole fresh cow | Milk, whole fresh cow | 75 | FAO |
| Cabbages and other brassicas | Others | Others | Cabbages and other brassicas | 19 | FAO |
| Cake, groundnuts | Others | Others | Groundnuts, with shell | 363 | FAO |
| Cake, sesame seed | Others | Others | Sesame seed | 376 | FAO |
| Cake, soybeans | Others | Others | Soybeans | 261 | FAO |
| Canary seed | Cereals (ex rice) | Others | Canary seed | 388 | FAO |
| Carrots and turnips | Others | Others | Carrots and turnips | 38 | FAO |
| Cashew nuts, shelled | Others | Others | Cashew nuts, with shell | 574 | FAO |
| Cashew nuts, with shell | Others | Others | Cashew nuts, with shell | 252 | FAO |
| Cashewapple | Others | Others | Cashewapple | 43 | FAO |
| Cassava | Others | Others | Cassava | 109 | FAO |
| Cassava dried | Others | Others | Cassava | 212 | FAO |
| Cauliflowers and broccoli | Others | Others | Cauliflowers and broccoli | 9 | FAO |
| Cereals, breakfast | Cereals (ex rice) | Others | Cereals, nes | 389 | FAO |
| Cereals, nes * | Cereals (ex rice) | Others | Cereals, nes | 340 | FAO |
| Cheese, processed | Milk | Milk, whole fresh cow | Milk, whole fresh cow | 103 | FAO |
| Cheese, sheep milk | Milk | Milk, whole fresh sheep | Milk, whole fresh sheep | 310 | FAO |
| Cheese, whole cow milk | Milk | Milk, whole fresh cow | Milk, whole fresh cow | 387 | FAO |
| Cherries | Others | Others | Cherries | 65 | FAO |

[6] USDA name: Broadbeans (fava beans), mature seeds, raw; USDA Data Type: SR Legacy.

| Commodity | Group | EI | Primary item | Calories (kcal/100g) | Source |
|---|---|---|---|---|---|
| Cherries, sour | Others | Others | Cherries, sour | 45 | FAO |
| Chestnut | Others | Others | Chestnut | 158 | FAO |
| Chick peas | Others | Others | Chick peas | 358 | FAO |
| Chillies and peppers, dry | Others | Others | Chillies and peppers, dry | 318 | FAO |
| Chillies and peppers, green | Others | Others | Chillies and peppers, green | 25 | FAO |
| Chocolate products nes | Others | Others | Cocoa, beans | 393 | FAO |
| Cinnamon (cannella) | Others | Others | Cinnamon (cannella) | 261 | FAO |
| Cloves | Others | Others | Cloves | 323 | FAO |
| Cocoa, beans | Others | Others | Cocoa, beans | 414 | FAO |
| Cocoa, butter | Others | Others | Cocoa, beans | 711 | FAO |
| Cocoa, paste | Others | Others | Cocoa, beans | 472 | FAO |
| Cocoa, powder & cake | Others | Others | Cocoa, beans | 261 | FAO |
| Coconuts | Others | Others | Coconuts | 184 | FAO |
| Coconuts, desiccated | Others | Others | Coconuts | 660 | FAO |
| Coffee, extracts | Others | Others | Coffee, green | 129 | FAO |
| Coffee, green | Others | Others | Coffee, green | 47 | FAO |
| Coffee, roasted | Others | Others | Coffee, green | 56 | FAO |
| Coffee, substitutes containing coffee | Others | Others | Coffee, green | 56 | FAO |
| Copra | Others | Others | Coconuts | 636 | FAO |
| Cottonseed | Others | Others | Cottonseed | 253 | FAO |
| Cranberries | Others | Others | Cranberries | 47 | FAO |
| Cream fresh | Milk | Milk, whole fresh cow | Milk, whole fresh cow | 195 | FAO |
| Cucumbers and gherkins | Others | Others | Cucumbers and gherkins | 13 | FAO |
| Currants | Others | Others | Currants | 59 | FAO |
| Dates | Others | Others | Dates | 156 | FAO |
| Eggplants (aubergines) | Others | Others | Eggplants (aubergines) | 21 | FAO |
| Eggs, dried | Eggs | Eggs, hen, in shell | Eggs, hen, in shell | 594 | FAO |
| Eggs, hen, in shell | Eggs | Eggs, hen, in shell | Eggs, hen, in shell | 139 | FAO |
| Eggs, liquid | Eggs | Eggs, hen, in shell | Eggs, hen, in shell | 158 | FAO |
| Eggs, other bird, in shell | Eggs | Eggs, hen, in shell | Eggs, other bird, in shell | 163 | FAO |
| Fat, camels | Others | Others | Meat, camel | 847 | FAO |
| Fat, cattle | Meat, ruminant | Meat, cattle | Fat, cattle | 847 | FAO |

| Commodity | Group | EI | Primary item | Calories (kcal/100g) | Source |
|---|---|---|---|---|---|
| Fat, liver prepared (foie gras) | Meat, poultry | Meat, chicken | Meat, goose and guinea fowl | 462 | FAO |
| Fat nes, prepared | Others | Others | Meat nes | 720 | FAO |
| Fat, pigs | Meat, pig | Meat, pig | Fat, pigs | 712 | FAO |
| Figs | Others | Others | Figs | 73 | FAO |
| Figs dried | Others | Others | Figs | 253 | FAO |
| Flour, cassava | Others | Others | Cassava | 338 | FAO |
| Flour, cereals | Cereals (ex rice) | Others | Cereals, nes | 364 | FAO |
| Flour, fonio | Cereals (ex rice) | Cereals excluding rice | Fonio | 355 | FAO |
| Flour, maize | Cereals (ex rice) | Cereals excluding rice | Maize | 363 | FAO |
| Flour, mixed grain | Cereals (ex rice) | Others | Grain, mixed | 364 | FAO |
| Flour, mustard | Others | Others | Mustard seed | 469 | FAO |
| Flour, potatoes | Others | Others | Potatoes | 355 | FAO |
| Flour, pulses | Others | Others | Pulses, nes | 340 | FAO |
| Flour, rice | Rice, paddy | Rice, paddy | Rice, paddy | 366 | FAO |
| Flour, roots and tubers nes | Others | Others | Roots and tubers nes | 282 | FAO |
| Flour, wheat | Cereals (ex rice) | Cereals excluding rice | Wheat | 364 | FAO |
| Fonio | Cereals (ex rice) | Others | Fonio | 338 | FAO |
| Fructose and syrup, other | Others | Others | Sugar crops nes | 368 | USDA[7] |
| Fruit, citrus nes * | Others | Others | Fruit, citrus nes | 26 | FAO |
| Fruit, cooked, homogenized preparations | Others | Others | Fruit, fresh nes | 59 | FAO |
| Fruit, dried nes | Others | Others | Fruit, fresh nes | 267 | FAO |
| Fruit, fresh nes | Others | Others | Fruit, fresh nes | 45 | FAO |
| Fruit, prepared nes | Others | Others | Fruit, fresh nes | 36 | FAO |
| Fruit, tropical fresh nes | Others | Others | Fruit, tropical fresh nes | 41 | FAO |
| Garlic | Others | Others | Garlic | 130 | FAO |
| Germ, maize | Cereals (ex rice) | Cereals excluding rice | Maize | 373 | FAO |

[7] USDA name: Sweeteners, tabletop, fructose, dry, powder; USDA Data Type: SR Legacy.

| Commodity | Group | EI | Primary item | Calories (kcal/100g) | Source |
|---|---|---|---|---|---|
| Ghee, buffalo milk | Milk | Milk, whole fresh buffalo | Milk, whole fresh buffalo | 873 | FAO |
| Ginger | Others | Others | Ginger | 347 | FAO |
| Glucose and dextrose | Others | Others | Sugar crops nes | 368 | FAO |
| Gooseberries | Others | Others | Gooseberries | 44 | FAO |
| Grain, mixed | Cereals (ex rice) | Others | Grain, mixed | 340 | FAO |
| Grapefruit (inc. pomelos) | Others | Others | Grapefruit (inc. pomelos) | 16 | FAO |
| Grapes | Others | Others | Grapes | 53 | FAO |
| Groundnuts, prepared | Others | Others | Groundnuts, with shell | 580 | FAO |
| Groundnuts, shelled | Others | Others | Groundnuts, with shell | 567 | FAO |
| Groundnuts, with shell * | Others | Others | Groundnuts, with shell | 414 | FAO |
| Hazelnuts, shelled | Others | Others | Hazelnuts, with shell | 632 | FAO |
| Hazelnuts, with shell | Others | Others | Hazelnuts, with shell | 291 | FAO |
| Honey, natural | Others | Others | Honey, natural | 298 | FAO |
| Hops | Others | Others | Hops | NA | NA |
| Ice cream and edible ice | Milk | Milk, whole fresh cow | Milk, whole fresh cow | 149 | FAO |
| Infant food | Cereals (ex rice) | Others | Cereals, nes | 368 | FAO |
| Juice, apple, concentrated | Others | Others | Apples | 166 | FAO |
| Juice, apple, single strength | Others | Others | Apples | 47 | FAO |
| Juice, citrus, concentrated | Others | Others | Fruit, citrus nes | 157 | FAO |
| Juice, citrus, single strength | Others | Others | Fruit, citrus nes | 47 | FAO |
| Juice, fruit nes | Others | Others | Fruit, fresh nes | 48 | FAO |
| Juice, grape | Others | Others | Grapes | 61 | FAO |
| Juice, grapefruit | Others | Others | Grapefruit (inc. pomelos) | 39 | FAO |
| Juice, grapefruit, concentrated | Others | Others | Grapefruit (inc. pomelos) | 146 | FAO |
| Juice, lemon, concentrated | Others | Others | Lemons and limes | 116 | FAO |
| Juice, lemon, single strength | Others | Others | Lemons and limes | 22 | FAO |
| Juice, orange, concentrated | Others | Others | Oranges | 159 | FAO |
| Juice, orange, single strength | Others | Others | Oranges | 42 | FAO |
| Juice, pineapple | Others | Others | Pineapples | 56 | FAO |

| Commodity | Group | EI | Primary item | Calories (kcal/100g) | Source |
|---|---|---|---|---|---|
| Juice, pineapple, concentrated | Others | Others | Pineapples | 179 | FAO |
| Juice, plum, concentrated | Others | Others | Plums and sloes | 215 | FAO |
| Juice, plum, single strength | Others | Others | Plums and sloes | 71 | FAO |
| Juice, tomato | Others | Others | Tomatoes | 17 | USDA[8] |
| Karite nuts (sheanuts) | Others | Others | Karite nuts (sheanuts) | 579 | FAO |
| Kiwi fruit | Others | Others | Kiwi fruit | 52 | FAO |
| Kola nuts | Others | Others | Kola nuts | 355 | FAO |
| Lactose | Milk | Milk, whole fresh cow | Milk, whole fresh cow | 387 | FAO |
| Lard | Meat, pig | Meat, pig | Fat, pigs | 902 | FAO |
| Leeks, other alliaceous vegetables | Others | Others | Leeks, other alliaceous vegetables | 37 | FAO |
| Lemons and limes | Others | Others | Lemons and limes | 15 | FAO |
| Lentils | Others | Others | Lentils | 346 | FAO |
| Lettuce and chicory | Others | Others | Lettuce and chicory | 12 | FAO |
| Linseed | Others | Others | Linseed | 498 | FAO |
| Macaroni | Cereals (ex rice) | Cereals excluding rice | Wheat | 367 | FAO |
| Maize | Cereals (ex rice) | Cereals excluding rice | Maize | 356 | FAO |
| Maize, green | Others | Others | Maize, green | 56 | FAO |
| Malt | Cereals (ex rice) | Cereals excluding rice | Barley | 368 | FAO |
| Mangoes, mangosteens, guavas | Others | Others | Mangoes, mangosteens, guavas | 45 | FAO |
| Maple sugar and syrups | Others | Others | Sugar crops nes | 348 | FAO |
| MatÃ© | Others | Others | MatÃ© | 40 | FAO |
| Meat, beef and veal sausages | Meat, ruminant | Meat, cattle | Meat, cattle | 313 | FAO |
| Meat, beef, preparations | Meat, ruminant | Meat, cattle | Meat, cattle | 233 | FAO |
| Meat, ass | Others | Others | Meat, ass | 94 | FAO |
| Meat, camel * | Others | Others | Meat, camel | 174 | FAO |
| Meat, cattle | Meat, ruminant | Meat, cattle | Meat, cattle | 225 | FAO |

[8] USDA name: Tomato juice, canned, without salt added; USDA Data Type: SR Legacy.

| Commodity | Group | EI | Primary item | Calories (kcal/100g) | Source |
|---|---|---|---|---|---|
| Meat, cattle, boneless (beef & veal) | Meat, ruminant | Meat, cattle | Meat, cattle | 150 | FAO |
| Meat, chicken | Meat, poultry | Meat, chicken | Meat, chicken | 122 | FAO |
| Meat, chicken, canned | Meat, poultry | Meat, chicken | Meat, chicken | 165 | FAO |
| Meat, dried nes | Others | Others | Meat nes | 250 | FAO |
| Meat, duck | Meat, poultry | Meat, chicken | Meat, duck | 291 | FAO |
| Meat, game | Others | Others | Meat, game | 104 | FAO |
| Meat, goat | Meat, ruminant | Meat, goat | Meat, goat | 123 | FAO |
| Meat, goose and guinea fowl | Meat, poultry | Meat, chicken | Meat, goose and guinea fowl | 301 | FAO |
| Meat, horse | Others | Others | Meat, horse | 85 | FAO |
| Meat nes | Others | Others | Meat nes | 126 | FAO |
| Meat, pig | Meat, pig | Meat, pig | Meat, pig | 326 | FAO |
| Meat, pig sausages | Meat, pig | Meat, pig | Meat, pig | 417 | FAO |
| Meat, pig, preparations | Meat, pig | Meat, pig | Meat, pig | 239 | FAO |
| Meat, pork | Meat, pig | Meat, pig | Meat, pig | 220 | FAO |
| Meat, rabbit | Others | Others | Meat, rabbit | 118 | FAO |
| Meat, sheep | Meat, ruminant | Meat, sheep | Meat, sheep | 263 | FAO |
| Meat, turkey | Meat, poultry | Meat, chicken | Meat, turkey | 126 | FAO |
| Melons, other (inc.cantaloupes) | Others | Others | Melons, other (inc.cantaloupes) | 17 | FAO |
| Milk, reconstituted | Milk | Milk, whole fresh cow | Milk, whole fresh cow | 61 | FAO |
| Milk, skimmed cow | Milk | Milk, whole fresh cow | Milk, whole fresh cow | 35 | FAO |
| Milk, skimmed dried | Milk | Milk, whole fresh cow | Milk, whole fresh cow | 362 | FAO |
| Milk, whole condensed | Milk | Milk, whole fresh cow | Milk, whole fresh cow | 321 | FAO |
| Milk, whole dried | Milk | Milk, whole fresh cow | Milk, whole fresh cow | 496 | FAO |
| Milk, whole evaporated | Milk | Milk, whole fresh cow | Milk, whole fresh cow | 134 | FAO |
| Milk, whole fresh buffalo * | Milk | Milk, whole fresh buffalo | Milk, whole fresh buffalo | 97 | FAO |
| Milk, whole fresh cow | Milk | Milk, whole fresh cow | Milk, whole fresh cow | 61 | FAO |
| Milk, whole fresh sheep | Milk | Milk, whole fresh sheep | Milk, whole fresh sheep | 94 | FAO |

| Commodity | Group | EI | Primary item | Calories (kcal/100g) | Source |
|---|---|---|---|---|---|
| Millet | Cereals (ex rice) | Cereals excluding rice | Millet | 340 | FAO |
| Molasses | Others | Others | Sugar beet and cane | 232 | FAO |
| Mushrooms and truffles | Others | Others | Mushrooms and truffles | 24 | FAO |
| Mushrooms, canned | Others | Others | Mushrooms and truffles | 24 | FAO |
| Mustard seed | Others | Others | Mustard seed | 469 | FAO |
| Nutmeg, mace and cardamoms | Others | Others | Nutmeg, mace and cardamoms | 525 | FAO |
| Nuts nes | Others | Others | Nuts nes | 262 | FAO |
| Nuts, prepared (exc. groundnuts) | Others | Others | Nuts nes | 615 | FAO |
| Oats | Cereals (ex rice) | Cereals excluding rice | Oats | 385 | FAO |
| Oats rolled | Cereals (ex rice) | Cereals excluding rice | Oats | 384 | FAO |
| Offals, edible, cattle | Meat, ruminant | Meat, cattle | Offals, edible, cattle | 105 | FAO |
| Offals, edible, goats | Meat, ruminant | Meat, goat | Offals, edible, goats | 117 | FAO |
| Offals, liver chicken | Meat, poultry | Meat, chicken | Offals, liver chicken | 125 | FAO |
| Offals, liver duck | Meat, poultry | Meat, chicken | Offals, liver duck | 136 | FAO |
| Offals, liver geese | Meat, poultry | Meat, chicken | Offals, liver geese | 133 | FAO |
| Offals, pigs, edible | Meat, pig | Meat, pig | Offals, pigs, edible | 113 | FAO |
| Offals, sheep,edible | Meat, ruminant | Meat, sheep | Offals, sheep,edible | 117 | FAO |
| Oil palm fruit * | Others | Others | Oil palm fruit | 158 | FAO |
| Oil, coconut (copra) | Others | Others | Coconuts | 884 | FAO |
| Oil, cottonseed | Others | Others | Cottonseed | 884 | FAO |
| Oils, fats of animal nes | Others | Others | Meat nes | 902 | FAO |
| Oil, groundnut | Others | Others | Groundnuts, with shell | 884 | FAO |
| Oil, linseed | Others | Others | Linseed | 884 | FAO |
| Oil, maize | Cereals (ex rice) | Cereals excluding rice | Maize | 884 | FAO |
| Oil, olive, virgin | Others | Others | Olives | 884 | FAO |
| Oil, palm | Others | Others | Oil palm fruit | 884 | FAO |
| Oil, palm kernel | Others | Others | Oil palm fruit | 884 | FAO |

| Commodity | Group | EI | Primary item | Calories (kcal/100g) | Source |
|---|---|---|---|---|---|
| Oil, poppy | Others | Others | Poppy seed | 884 | FAO |
| Oil, rapeseed | Others | Others | Rapeseed | 884 | FAO |
| Oil, rice bran | Rice, paddy | Rice, paddy | Rice, paddy | 884 | FAO |
| Oil, safflower | Others | Others | Safflower seed | 884 | FAO |
| Oil, sesame | Others | Others | Sesame seed | 884 | FAO |
| Oil, soybean | Others | Others | Soybeans | 884 | FAO |
| Oil, sunflower | Others | Others | Sunflower seed | 884 | FAO |
| Oil, vegetable origin nes | Others | Others | Oilseeds nes | 884 | FAO |
| Oilseeds nes | Others | Others | Oilseeds nes | 387 | FAO |
| Olives | Others | Others | Olives | 175 | FAO |
| Olives preserved | Others | Others | Olives | 109 | FAO |
| Onions, dry | Others | Others | Onions, dry | 31 | FAO |
| Onions, shallots, green | Others | Others | Onions, shallots, green | 24 | FAO |
| Oranges | Others | Others | Oranges | 34 | FAO |
| Papayas | Others | Others | Papayas | 26 | FAO |
| Pastry | Cereals (ex rice) | Cereals excluding rice | Wheat | 369 | FAO |
| Peaches and nectarines | Others | Others | Peaches and nectarines | 33 | FAO |
| Peanut butter | Others | Others | Groundnuts, with shell | 589 | FAO |
| Pears | Others | Others | Pears | 54 | FAO |
| Peas, dry | Others | Others | Peas, dry | 346 | FAO |
| Peas, green | Others | Others | Peas, green | 31 | FAO |
| Pepper (piper spp.) | Others | Others | Pepper (piper spp.) | 276 | FAO |
| Peppermint | Others | Others | Peppermint | 70 | USDA[9] |
| Persimmons | Others | Others | Persimmons | 82 | FAO |
| Pineapples | Others | Others | Pineapples | 26 | FAO |
| Pineapples canned | Others | Others | Pineapples | 78 | FAO |
| Pistachios | Others | Others | Pistachios | 289 | FAO |
| Plantains and others | Others | Others | Plantains and others | 75 | FAO |
| Plums and sloes | Others | Others | Plums and sloes | 52 | FAO |
| Plums dried (prunes) | Others | Others | Plums and sloes | 208 | FAO |
| Popcorn | Cereals (ex rice) | Cereals excluding rice | Maize | 356 | FAO |
| Poppy seed | Others | Others | Poppy seed | 533 | FAO |
| Potatoes | Others | Others | Potatoes | 67 | FAO |
| Potatoes, frozen | Others | Others | Potatoes | 73 | FAO |
| Pulses, nes * | Others | Others | Pulses, nes | 340 | FAO |

[9] USDA name: Peppermint, fresh; USDA Data Type: SR Legacy.

| Commodity | Group | EI | Primary item | Calories (kcal/100g) | Source |
|---|---|---|---|---|---|
| Pumpkins, squash and gourds | Others | Others | Pumpkins, squash and gourds | 19 | FAO |
| Quinces | Others | Others | Quinces | 35 | FAO |
| Quinoa | Others | Others | Quinoa | 342 | FAO |
| Raisins | Others | Others | Grapes | 299 | FAO |
| Rapeseed | Others | Others | Rapeseed | 494 | FAO |
| Rice - total (Rice milled equivalent) | Rice, paddy | Rice, paddy | Rice, paddy | 359 | FAO |
| Rice, broken | Rice, paddy | Rice, paddy | Rice, paddy | 360 | FAO |
| Rice, husked | Rice, paddy | Rice, paddy | Rice, paddy | 357 | FAO |
| Rice, milled | Rice, paddy | Rice, paddy | Rice, paddy | 360 | FAO |
| Rice, paddy * | Rice, paddy | Rice, paddy | Rice, paddy | 280 | FAO |
| Roots and tubers nes | Others | Others | Roots and tubers nes | 91 | FAO |
| Rye | Cereals (ex rice) | Cereals excluding rice | Rye | 319 | FAO |
| Safflower seed * | Others | Others | Safflower seed | 314 | FAO |
| Sesame seed | Others | Others | Sesame seed | 573 | FAO |
| Sorghum | Cereals (ex rice) | Cereals excluding rice | Sorghum | 343 | FAO |
| Soya curd | Others | Others | Soybeans | 58 | FAO |
| Soya paste | Others | Others | Soybeans | 114 | FAO |
| Soya sauce | Others | Others | Soybeans | 56 | FAO |
| Soybeans | Others | Others | Soybeans | 335 | FAO |
| Spices nes | Others | Others | Spices nes | 337 | FAO |
| Spinach | Others | Others | Spinach | 16 | FAO |
| Starch, cassava | Others | Others | Cassava | 362 | FAO |
| Strawberries | Others | Others | Strawberries | 28 | FAO |
| Sugar beet | Others | Others | Sugar beet | 70 | FAO |
| Sugar beet and cane * | Others | Others | Sugar beet and cane | 50 | Calculated |
| Sugar confectionery | Others | Others | Sugar crops nes | 310 | FAO |
| Sugar crops nes | Others | Others | Sugar crops nes | 390 | FAO |
| Sugar flavoured | Others | Others | Sugar crops nes | 310 | FAO |
| Sugar non-centrifugal | Others | Others | Sugar beet and cane | 351 | FAO |
| Sugar Raw Centrifugal | Others | Others | Sugar beet and cane | 373 | FAO |
| Sugar refined | Others | Others | Sugar beet and cane | 387 | FAO |
| Sugar nes | Others | Others | Sugar crops nes | 310 | FAO |

| Commodity | Group | EI | Primary item | Calories (kcal/100g) | Source |
|---|---|---|---|---|---|
| Sunflower seed | Others | Others | Sunflower seed | 308 | FAO |
| Sweet corn frozen | Others | Others | Maize, green | 54 | FAO |
| Sweet corn prep or preserved | Others | Others | Maize, green | 77 | FAO |
| Sweet potatoes | Others | Others | Sweet potatoes | 92 | FAO |
| Tangerines, mandarins, clementines, satsumas | Others | Others | Tangerines, mandarins, clementines, satsumas | 32 | FAO |
| Tea | Others | Others | Tea | 40 | FAO |
| Tea, mate extracts | Others | Others | Tea | 18 | FAO |
| Tomatoes | Others | Others | Tomatoes | 17 | FAO |
| Tomatoes, paste | Others | Others | Tomatoes | 84 | FAO |
| Tomatoes, peeled | Others | Others | Tomatoes | 19 | FAO |
| Triticale | Cereals (ex rice) | Others | Triticale | 327 | FAO |
| Vanilla | Others | Others | Vanilla | 288 | USDA[10] |
| Vegetables in vinegar | Others | Others | Vegetables, fresh nes | 29 | FAO |
| Vegetables, canned nes | Others | Others | Vegetables, fresh nes | 36 | FAO |
| Vegetables, dehydrated | Others | Others | Vegetables, fresh nes | 341 | FAO |
| Vegetables, dried nes | Others | Others | Vegetables, fresh nes | 176 | FAO |
| Vegetables, fresh nes | Others | Others | Vegetables, fresh nes | 22 | FAO |
| Vegetables, fresh or dried products nes | Others | Others | Vegetables, fresh nes | 22 | FAO |
| Vegetables, frozen | Others | Others | Vegetables, fresh nes | 71 | FAO |
| Vegetables, homogenized preparations | Others | Others | Vegetables, fresh nes | 41 | FAO |
| Vegetables, preserved nes | Others | Others | Vegetables, fresh nes | 38 | FAO |
| Vegetables, preserved, frozen | Others | Others | Vegetables, fresh nes | 22 | FAO |
| Vegetables, temporarily preserved | Others | Others | Vegetables, fresh nes | 65 | FAO |
| Vermouths & similar | Others | Others | Grapes | 137 | FAO |
| Vetches | Others | Others | Vetches | 325 | FAO |

[10] USDA name: Vanilla extract; USDA Data Type: SR Legacy.

| Commodity | Group | EI | Primary item | Calories (kcal/100g) | Source |
|---|---|---|---|---|---|
| Wafers | Cereals (ex rice) | Others | Cereals, nes | 439 | FAO |
| Walnuts, shelled | Others | Others | Walnuts, with shell | 642 | FAO |
| Walnuts, with shell | Others | Others | Walnuts, with shell | 289 | FAO |
| Watermelons | Others | Others | Watermelons | 17 | FAO |
| Wheat | Cereals (ex rice) | Cereals excluding rice | Wheat | 334 | FAO |
| Whey, condensed | Milk | Milk, whole fresh cow | Milk, whole fresh cow | 26 | FAO |
| Whey, dry | Milk | Milk, whole fresh cow | Milk, whole fresh cow | 346 | FAO |
| Wine | Others | Others | Grapes | 68 | FAO |
| Yoghurt | Milk | Milk, whole fresh cow | Milk, whole fresh cow | 61 | FAO |
| Yoghurt, concentrated or not | Milk | Milk, whole fresh cow | Milk, whole fresh cow | 82 | FAO |
