# Supplementary material for: Adjusting agricultural emissions for trade matters for climate change mitigation
Source: Nat Commun. 2022 Jun 9;13:3024. doi: 10.1038/s41467-022-30607-x (PMC9184627; doi:10.1038/s41467-022-30607-x)
Supplement: Supplementary file 3 — Reporting Summary [file 41467_2022_30607_MOESM3_ESM.pdf]

nature portfolio

Corresponding author(s): Prajal Pradhan

Last updated by author(s): Apr 13, 2022

Reporting Summary

Nature Portfolio wishes to improve the reproducibility of the work that we publish. This form provides structure for consistency and transparency in reporting. For further information on Nature Portfolio policies, see our Editorial Policies and the Editorial Policy Checklist.

Statistics

For all statistical analyses, confirm that the following items are present in the figure legend, table legend, main text, or Methods section.

n/a	Confirmed	
☒	☐	The exact sample size (*n*) for each experimental group/condition, given as a discrete number and unit of measurement
☒	☐	A statement on whether measurements were taken from distinct samples or whether the same sample was measured repeatedly
☒	☐	The statistical test(s) used AND whether they are one- or two-sided<br>*Only common tests should be described solely by name; describe more complex techniques in the Methods section.*
☒	☐	A description of all covariates tested
☒	☐	A description of any assumptions or corrections, such as tests of normality and adjustment for multiple comparisons
☐	☒	A full description of the statistical parameters including central tendency (e.g. means) or other basic estimates (e.g. regression coefficient) AND variation (e.g. standard deviation) or associated estimates of uncertainty (e.g. confidence intervals)
☒	☐	For null hypothesis testing, the test statistic (e.g. *F*, *t*, *r*) with confidence intervals, effect sizes, degrees of freedom and *P* value noted<br>*Give P values as exact values whenever suitable.*
☒	☐	For Bayesian analysis, information on the choice of priors and Markov chain Monte Carlo settings
☒	☐	For hierarchical and complex designs, identification of the appropriate level for tests and full reporting of outcomes
☒	☐	Estimates of effect sizes (e.g. Cohen's *d*, Pearson's *r*), indicating how they were calculated

Our web collection on statistics for biologists contains articles on many of the points above.

Software and code

Policy information about availability of computer code

Data collection	No software or code was used for data collection.
Data analysis	The data analysis was carried out using open source R version 3.6.3 (2020-02-29) and RStudio (Version 1.2.5033).

For manuscripts utilizing custom algorithms or software that are central to the research but not yet described in published literature, software must be made available to editors and reviewers. We strongly encourage code deposition in a community repository (e.g. GitHub). See the Nature Portfolio guidelines for submitting code & software for further information.

Data

Policy information about availability of data

All manuscripts must include a data availability statement. This statement should provide the following information, where applicable:

- Accession codes, unique identifiers, or web links for publicly available datasets
- A description of any restrictions on data availability
- For clinical datasets or third party data, please ensure that the statement adheres to our policy

All processed data, country data, and food data are available in Figshare under the following link: doi.org/10.6084/m9.figshare.19583194. The original FAOSTAT data are available in the FAOSTAT open-source database under the following link: https://www.fao.org/faostat/en/#data. The data from Romanello et al. (2021) was obtained upon request from the authors. Food caloric content values were obtained from FAO (2019): http://www.fao.org/economic/the-statistics-division-ess/publications-studies/publications/nutritive-factors/en/; and USDA (2018): https://fdc.nal.usda.gov/index.html/.

Field-specific reporting

Please select the one below that is the best fit for your research. If you are not sure, read the appropriate sections before making your selection.

☐ Life sciences ☐ Behavioural & social sciences ☒ Ecological, evolutionary & environmental sciences

For a reference copy of the document with all sections, see nature.com/documents/nr-reporting-summary-flat.pdf

Ecological, evolutionary & environmental sciences study design

All studies must disclose on these points even when the disclosure is negative.

Study description	This study explores recent trends in trade-adjusted agricultural emissions of food items at the global, regional, and national levels, which we found are largely dependent on a country's consumption patterns and their agricultural emission intensities relative to their trading partners'.
Research sample	We chose to use the data from FAOSTAT as our main data for analysis, owing to its comprehensive coverage of countries (over 211 countries), years (1986 to present), and food items (443 in total, based on FAOSTAT trade data). We thus believe that the dataset is representative.
Sampling strategy	This study aims to cover all countries and food items where data is available. Hence, sampling strategy and size do not apply to this study.
Data collection	Data was downloaded in CSV format from the open-source FAOSTAT database (https://www.fao.org/faostat/en/#data).
Timing and spatial scale	The data used for this study are at country scale with annual values from 1986 to 2017 (i.e., the time frame available at the time of study)
Data exclusions	No data were excluded.
Reproducibility	The code and data used for this study are available and described in further detail in Figshare under the following link: doi.org/10.6084/m9.figshare.19583194
Randomization	This study aims to cover all countries and food items where data is available. Hence, randomization does not apply to our study design.
Blinding	This study is based on freely available secondary data. Thus, blinding was not applicable to the study.

Did the study involve field work? ☐ Yes ☒ No

Reporting for specific materials, systems and methods

We require information from authors about some types of materials, experimental systems and methods used in many studies. Here, indicate whether each material, system or method listed is relevant to your study. If you are not sure if a list item applies to your research, read the appropriate section before selecting a response.

Materials & experimental systems

n/a	Involved in the study	
☒	☐	Antibodies
☒	☐	Eukaryotic cell lines
☒	☐	Palaeontology and archaeology
☒	☐	Animals and other organisms
☒	☐	Human research participants
☒	☐	Clinical data
☒	☐	Dual use research of concern

Methods

n/a	Involved in the study	
☒	☐	ChIP-seq
☒	☐	Flow cytometry
☒	☐	MRI-based neuroimaging
